# Type of Ground Surface during Plyometric Training Affects the Severity of Exercise-Induced Muscle Damage

**DOI:** 10.3390/sports4010015

**Published:** 2016-03-01

**Authors:** Hamid Arazi, Roger Eston, Abbas Asadi, Behnam Roozbeh, Alireza Saati Zarei

**Affiliations:** 1 Department of Exercise Physiology, Faculty of Sport Sciences, University of Guilan, Rasht 1438, Iran; abbas_asadi1175@yahoo.com (A.A.); behnam.roozbeh@yahoo.com (B.R.); alisaati7@yahoo.com (A.S.Z.); 2 Alliance for Research in Exercise, Nutrition and Activity; School of Health Sciences, University of South Australia, Adelaide 5000, Australia; Roger.Eston@unisa.edu.au; 3 Roudbar Branch, Islamic Azad University, Roudbar, Iran

**Keywords:** plyometric exercise, aquatic setting, sand surface, muscle soreness

## Abstract

The purpose of this study was to compare the changes in the symptoms of exercise-induced muscle damage from a bout of plyometric exercise (PE; 10 × 10 vertical jumps) performed in aquatic, sand and firm conditions. Twenty-four healthy college-aged men were randomly assigned to one of three groups: Aquatic (AG, *n* = 8), Sand (SG, *n* = 8) and Firm (FG, *n* = 8). The AG performed PE in an aquatic setting with a depth of ~130 cm. The SG performed PE on a dry sand surface at a depth of 20 cm, and the FG performed PE on a 10-cm-thick wooden surface. Plasma creatine kinase (CK) activity, delayed onset muscle soreness (DOMS), knee range of motion (KROM), maximal isometric voluntary contraction (MIVC) of the knee extensors, vertical jump (VJ) and 10-m sprint were measured before and 24, 48 and 72 h after the PE. Compared to baseline values, FG showed significantly (*p* < 0.05) greater changes in CK, DOMS, and VJ at 24 until 48 h. The MIVC decreased significantly for the SG and FG at 24 until 48 h post-exercise in comparison to the pre-exercise values. There were no significant (*p* > 0.05) time or group by time interactions in KROM. In the 10-m sprint, all the treatment groups showed significant (*p* < 0.05) changes compared to pre-exercise values at 24 h, and there were no significant (*p* > 0.05) differences between groups. The results indicate that PE in an aquatic setting and on a sand surface induces less muscle damage than on a firm surface. Therefore, training in aquatic conditions and on sand may be beneficial for the improvement of performance, with a concurrently lower risk of muscle damage and soreness.

## 1. Introduction

Lower body plyometric training involves performing repeated consecutive jumping-type exercises with one’s own body weight against gravity. Plyometric training is widely used to enhance the ability of skeletal muscles to generate power. The method involves a repeated series of bouts with each comprising a rapid deceleration of the body, followed immediately by a brief transition phase and rapid acceleration in the opposite direction [1,2,3]. This rapid combination of eccentric and concentric muscular activity involves the stretch-shortening cycle (SSC), which provides a physiological advantage in that the muscular force developed during the concentric phase is potentiated by the preceding eccentric contraction [1]. The use of plyometric training results in the improvement of vertical jumping ability (VJ) [2,3,4], strength [2,4], agility [3], sprint [3,4], balance [5] and running economy [6]. However, the eccentric contractions involved in plyometrics have been shown to induce skeletal-muscle trauma resulting in exercise-induced muscle damage (EIMD) [5,6,7,8,9,10,11]. Indeed, plyometric exercise (PE) in the form of vertical countermovement jumps on a solid surface is frequently used in studies as a means of inducing EIMD in the knee extensors [9,10,11,12,13]. Frequently observed symptoms of EIMD include muscle soreness [4,10,13], an increase in plasma creatine kinase activity (CK) [12,14,15], loss of strength [10,13] and power [5,14] and a reduction in joint range of motion (ROM) [15,16,17,18,19]. These effects begin approximately 6 h after exercise, peak at 24–72 h and subside four to seven days after exercise [11,12,13,14,19].

An important consideration regarding the impact of PE on EIMD is the nature of the training surface. Implementation of plyometric training in an aquatic setting has been observed to induce less EIMD and muscle soreness than plyometric training on a firm surface, but with the same improvements in muscular performance [4,20]. An aquatic setting provides a low-impact medium that produces less strain on muscles, bones and connective tissue. An aquatic environment also provides buoyancy which reduces weight-bearing stress on the limbs and thus reduces the impact forces and the potential trauma to the joints, thereby providing a therapeutic modality for reducing muscle soreness and pain [2,4,20]. Less EIMD is observed after similar plyometric activity on a sand surface compared to a firm, wooden surface. Robinson *et al.* [4] attributed the lower muscle soreness in the aquatic *versus* the land plyometric group to less strain on the musculoskeletal system. Similarly, as a sand surface is associated with a greater degree of shock absorbance and lower stress to soft tissue and bones of the lower limbs during PE [20,21]. The compliant nature of the sand surface plays a critical role for decreasing landing force and the absorptive qualities of sand are likely to decrease stress on the lower limbs, resulting in less muscle damage than that obtained on a firm surface [19,21,22].

Although several studies have explored how the impact of plyometric training on EIMD is affected by the nature of the landing and take-off surface, such as sand against grass [22], sand against wood [19] and aquatic against wood [4], no study has directly compared aquatic, sand and firm conditions in relation to muscle damage and the sense of effort in performing a similar number of plyometric jumps. It is important to understand the differences in response to PE among aquatic, sand and firm surfaces, since many assumptions have been made from studies using different surfaces that have reported the important effects of landing surface for reducing muscle damage with regard to the nature of surface. The present study used consecutive vertical jumps performed on aquatic, sand and firm surfaces as a model to investigate the influence of surface on EIMD. We hypothesized that PE would disturb indirect markers of muscle damage, increase muscle soreness and plasma creatine kinase activity for days after exercise and that this disturbance would be attenuated for the aquatic and sand surfaces.

## 2. Methods

### 2.1. Study Design

Three groups of male college students performed a series of vertical jumps (VJ) on either a dry sand surface, a dry wooden surface or a ceramic tiled surface in aquatic conditions. The aquatic group (AG) performed PE in a swimming pool with water at a depth of 130 cm (chest-deep) and a temperature of 28 °C; the sand group (SG) performed PE on a dry surface of 20-cm-deep sand and the firm surface group (FG) performed PE on a 10-cm-thick wood surface. Indirect markers of muscle damage (plasma creatine kinase activity, range of motion, isometric strength, muscle soreness, vertical jump height and sprint speed) were assessed at baseline, and 24, 48 and 72 h after the PE protocol. All risks associated with the experimental procedures were explained prior to involvement in the study and each participant was asked to complete a written informed consent form and a medical health questionnaire before assessment. Procedures and the study were conducted in accordance with ethical standards in sport and exercise science research [23] and approved by the Local Ethics Committee.

### 2.2. Participants

Twenty-four healthy untrained young men, who were familiar with plyometric exercise training but who had not performed this type of exercises for at least six months prior to the study, volunteered for the study. All participants were free from injury. Prior to recruitment, participants were asked about their activity levels. Those who performed any lower extremity resistive exercise/eccentric resistive exercise were excluded from the study. Prior to the initial testing, each participant was familiarized with the testing protocol and completed a full practice testing session and plyometric exercise. After this familiarization session, each participant was tested on four separate occasions using identical protocols at 24 h before starting exercise, and 24, 48 and 72 h post plyometric exercise. After baseline testing, participants were randomly assigned to three treatment groups; AG (*N* = 8, age 20.2 ± 0.4 y, height 180.5 ± 4.2 cm, body mass 73.3 ± 7.1 kg), SG (*N* = 8, age 20.3 ± 0.7 y, height 177.8 ± 6.6 cm, body mass 67.8 ± 9.5 kg), and FG (*N* = 8, age 20.7 ± 0.4 y, height 180.1 ± 5.5 cm, body mass 71.7 ± 9.6 kg). *A priori* calculations of statistical power indicated that this sample size was appropriate to satisfy power at or above 80% [24]. Participants performed PE barefoot, because (a) most sports activities on sand and in aquatic setting are performed without shoes, and (b) it is easier to compare differences in responses among conditions by eliminating the effect of shoes. The investigators carefully supervised the exercise to eliminate the risks of unexpected injury. Participants abstained from any strenuous physical activity for at least 15 days before and after the experimental period. Participants were instructed to maintain their normal eating pattern for two weeks before data collection and during the investigation. Participants were not taking any medication or dietary supplements with anti-inflammatory action for six months before the study.

### 2.3. Plyometric Exercise Protocol

All participants performed 10 sets of 10 maximal vertical jumps, interspersed with a one-minute recovery between each set. Immediately prior to starting the exercise, a maximal vertical jump was performed with a chalk mark made by the fingertips at the highest point of the jump. The participants were instructed to perform each jump with maximal effort to reach the target height and the authors encouraged the participants to reach the mark. On landing, participants were instructed to adopt a knee joint angle of ~90°. This protocol has been used successfully to induce EIMD in previous studies [11,12].

### 2.4. Dependent Variables

Indirect markers of muscle damage were plasma creatine kinase (CK) activity, delayed onset muscle soreness (DOMS), knee flexion range of motion (KROM), maximal isometric voluntary contraction (MIVC) of the knee extensors, vertical jump height (VJ) and 10-m sprint time. The rating of perceived exertion (RPE) was also assessed after each set of the plyometric exercise. One week prior to commencement of the study, the participants attended the laboratory for the familiarization of testing procedures. During this session the participants performed three to four trials of each test to eliminate any potential learning effect.

### 2.5. Plasma Creatine Kinase Activity (CK)

Blood samples were drawn (10 mL) from the antecubital vein into plain evacuated test tubes. The blood was allowed to clot at room temperature for 30 min and centrifuged at 1500× g for 10 min. The serum layer was removed and frozen at −20 °C in multiple aliquots for further analyses. Plasma CK activity was determined spectrophotometrically (Milton Roy, Spectronic 401, Rochester, NY, USA) in duplicates using a commercially available kit (Autoanalyzer, Technicon RA-1000, Clackamas, OR, USA). The normal reference range of CK activity for men using this method is 40−195 U/L. The intra-assay coefficient of variation was 8.5% for CK.

### 2.6. Delayed Onset Muscle Soreness (DOMS)

Each participant was asked to indicate muscle soreness (DOMS) of the knee extensors using a visual analogue scale. The scale was numbered from 1 to 10 (on the reverse side of the sliding scale) with 1 indicating no muscle soreness and 10 signifying that the muscle was very, very sore to move. With hands on hips and squatting to an approximate knee angle of 90°, volunteers were asked to indicate the level of perceived soreness based on the rating scale. This corresponded to the location of perceived muscle soreness on the continuum. This technique has been used successfully in previous studies [11,16]. The reliability coefficient for a repetitive measurement in DOMS was 0.98.

### 2.7. Knee Flexion Range of Motion (KROM)

The knee flexion range of motion (KROM) testing protocol has been previously described by Clarkson [25]. From a prone-lying position, participants were asked to perform full active flexion of the knee of the right leg. The knee joint angle was measured between the lateral epicondyle of the femur, lateral malleous of the fibula and greater trochanter using a goniometer (Lafayette Instrument Company, Lafayette, IN, USA). These bony landmarks were marked on the initial day of testing with a semi-permanent pen to ensure consistency on subsequent days. The mean of two measurements was used for data analysis. Reliability coefficient for repetitive measurements in KROM was 0.99.

### 2.8. Maximal Isometric Voluntary Contraction (MIVC)

Maximal isometric voluntary contraction (MIVC) of the quadriceps was assessed at a joint angle of 70° of flexion using a JAMAR^®^ dynamometer (Back-Leg-Chest Dynamometer, Model 68815, Gays Mills, WI, USA) because Byrne *et al.* [26] suggest the optimal knee angle for isometric knee extensor torque is 60°–80°. The joint position was marked on the initial day of testing with a semi-permanent pen to ensure consistency during subsequent testing sessions. Participants were given a total of two trials with maximal effort and each repetition lasted 3 s and was interspersed with 60 s of rest. The best value was collected for the further analyses [15,19]. Reliability coefficient for repetitive measurements in MIVC was 0.93.

### 2.9. Vertical Jump (VJ)

The vertical jump (VJ) was assessed using a Vertec (Power Systems, Knoxville, Tennessee, TN, USA). The Vertec was adjusted to match the height of the individual participant by having him stand with the dominant side to the base of the testing device. The dominant hand was raised and the Vertec was adjusted so that the hand was the appropriate distance away from the marker based on markings on the device itself. VJ was performed from a squatting position with a knee joint angle of ~90°. Participants were asked to hold this position for three seconds, then jump upward in an attempt to reach the maximal height with no countermovement after a verbal command of “Go”. Each test was performed twice, and the best value of the two measurements was used for the analysis [9]. Reliability coefficient for repetitive measurements in VJ was 0.95.

### 2.10. The 10-m Sprint

Participants performed two sets of a single 10-m sprint from a standing start on an indoor track with a 3-min recovery. Prior to commencing the test, participants performed two sub-maximal jogs. Sprint time was recorded using two electronic photo cells positioned at 0 (start) and 10 m (JBL Systems, Oslo, Norway). Time for sprint performance was recorded to the nearest 0.01 s via telemetry to a handheld system. The fastest time recorded was used for analysis [11]. Reliability coefficient for repetitive measurements in 10-m sprint was 0.96.

### 2.11. Rating of Perceived Exertion (RPE)

The rating of perceived exertion (RPE) using the Borg 6-20 RPE Scale [27] was recorded immediately following each set of jumps. After the final repetition of each set, participants were reminded to think about feelings of exertion in the active muscle group, in accordance with previous procedures [28].

### 2.12. Statistical Analysis

Data are presented as mean ± SD. Data normality was checked and verified with the Shapiro-Wilk test. As the CK activity data were found not to be normally distributed, the values were log-transformed before statistical analysis [11,18,29]. Following transformation the CK activity data were normally distributed. A repeated-measures ANOVA (3 × 4, group × time) was used to analyze data (SPSS 16.0). Assumptions of sphericity were assessed using Mauchly’s test of sphericity, with any violations adjusted by use of the Greenhouse-Geisser(GG) correction. To assess changes in RPE between sets, a 3 (group) × 10 (set) ANOVA was applied to the data. When a significant *F* value was achieved, a Tukey *post hoc* test was used to detect differences in the measures. Significance was set at *p* ≤ 0.05.

## 3. Results

### 3.1. CK Activity

**Figure 1:**
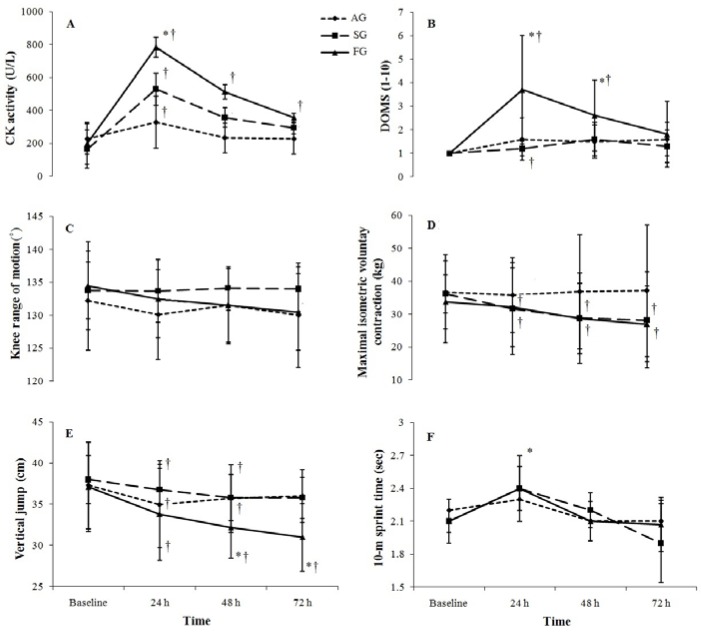
Mean ± SD for creatine kinase (CK) activity (**A**), delayed onset muscle soreness (DOMS) (**B**), knee flexion range of motion (**C**), maximal isometric voluntary contraction (**D**), vertical jump (**E**) and 10-m sprint (**F**) at baseline and 24–72 h following 100 plyometric jumps. AG: Aquatic group; SG: Sand group; FG: Firm group. *: Significant difference compared to AG and SG (*p* ≤ 0.05); †: Significant difference compared to baseline (*p* ≤ 0.05).

There was a significant increase in CK activity after the plyometric exercise protocol for all groups, with CK values peaking at 24 h for each group (*p* < 0.01). There was a significant group × time interaction, which indicated a greater increase in CK for FG than for SG and AG (*p* ≤ 0.05) at 24 h, which remained higher than baseline at 72 h (Figure 1A).

### 3.2. Muscle Soreness

There was a significant group × time interaction which indicated significantly greater soreness in the FG compared to the AG and SG at 24 and 48 h post-exercise (*p* ≤ 0.05). Compared with baseline data, muscle soreness was greater at 24 and 48 h for FG and at 24 h for SG. There was no significant change in soreness for the AG (Figure 1B).

### 3.3. Knee Range of Motion (KROM)

No statistically significant changes in knee range of motion were observed after plyometric exercises in the experimental groups (*p* > 0.05) (Figure 1C).

### 3.4. Maximal Isometric Voluntary Contraction (MIVC)

No statistically significant group × time interactions in muscle strength were observed after plyometric exercises, although changes across time were significance for the FG and SG (*p* ≤ 0.05) (Figure 1D).

### 3.5. Vertical Jump

All treatment groups showed a significant decrease in VJ at 24 and 48 h post-exercise (*p* < 0.01). There was also a significant group × time interaction (*p* < 0.01). Follow-up analysis indicated that the decrease in vertical jump height was similar for the AG and SG, whereas the decrease in VJ was greater for the FG at 48 h and remained lower at 72 h following the plyometric exercise protocol (Figure 1E).

### 3.6. 10-m Sprint Times

The peak running time over 10 m was significantly higher following plyometric exercises in all groups (*p* < 0.01). There was no significant group × time interaction. *Post hoc* analysis showed that the sprint time at 24 hours was significantly higher than baseline values before returning to baseline levels at 48 h (Figure 1F).

## 4. Rating of Perceived Exertion (RPE)

Table 1 shows the changes in RPE following each set of VJ exercises. The RPE increased progressively throughout the plyometric VJ testing (*p* < 0.01). There was no significant group main effect, and no group × set interaction. *Post hoc* analysis showed that set 4 was perceived to be harder than set 1, and set 5 harder than set 2, and sets 9 and 10 were harder than set 7.

## 5. Discussion

Plyometrics is a form of explosive physical training that is used to enhance power output, force production and velocity [2,3,4]. This type of training has been shown to induce skeletal-muscle damage [7,8,9,10,11,12,13,14]. Although a number of studies have assessed the effects of plyometric exercise on indirect indices of muscle damage (*i.e.*, VJ, strength, sprint, ROM and agility) [7,8,9,10,11,12,13,14,16,18,19], limited knowledge exists regarding how the extent of damage and post-exercise recovery is moderated by the type of surface. To the authors’ knowledge, this is the first study that has directly compared the symptoms of EIMD between aquatic, sand and firm surface conditions. Overall, we found that the symptoms of EIMD were greater for the participants in the firm surface group (FG), while the changes in indices of muscle damage were generally significantly smaller for the AG.

## 6. Evidence of Muscle Damage

This research has shown that an intense, acute bout of PE (10 × 10 VJs) increased plasma CK activity and DOMS at 24–72 h post-exercise for the FG and the SG. The changes in CK were consistent with those observed following plyometric exercises with values peaking at 24 h after the damaging exercise in the firm and sand conditions [17,30,31]. Perceived soreness (DOMS) was also increased above the baseline value following muscle-damaging exercise, with peak values occurring at 48 h for participants in the SG and at 24 h for the FG. These findings mirror those of previous research, which has demonstrated increases in muscle soreness peaking between 24 and 48 h [9,12,14,30,31]. Previous investigations have suggested that PE on a firm wooden surface may induce muscle damage because of the forces produced during ground impact and the associated eccentric contractions [14,16] and this damage is greater in the type II muscle and Z-line [30,31]. To resist the impact of landing, the quadriceps muscles perform an eccentric action that involves a counter-extension movement to absorb kinetic energy. During the negative phase of PE, eccentric activation produces higher tension per cross-sectional area of active muscle mass compared with concentric actions, resulting in significant structural muscle damage [16]. Compared with the aquatic group, the FG indicated higher perception of muscle soreness (~4 *vs.* 1) and a greater level of plasma CK activity. These results provide further confirmation that PE in waist-deep water provides a safer environment and reduces impact on the joints and muscles due to the buoyancy effect which appears to reduce the extent of musculoskeletal trauma and damage [4,19].

In addition, we observed a limited development of muscle soreness for the SG at 48 h. The lack of change in muscle soreness may be associated with the execution of the PE on a soft surface (sand and/or aquatic) because it is known that performing plyometrics on a soft surface induces less muscle damage than a firm surface [4,19]. The compliance and instability of the sand results in a reduction of maximum force and landing force during plyometrics, resulting in less muscle damage and soreness compared to the firm surface condition [21,22]. On the other hand, performing PE in an aquatic setting could provide a non-impact medium that reduces stress on the lower limbs, providing a therapeutic modality for reducing muscle soreness and damage [2,4,20]. Similarly, the absorptive qualities of the sand surface during PE decreased stress on the lower limbs, resulting in less muscle damage than that on firm surface [19,21,22].

## 7. Muscle Function during Recovery from Plyometric Exercise

Although several studies have examined the effects of PE on muscle function, to our knowledge, this is the first attempt to examine the effects of different surfaces during plyometrics on muscle function. In this study, we measured muscle function using knee range of motion (KROM), maximal isometric voluntary contraction (MIVC), vertical jump (VJ) and 10-m sprint time. The decreases in MIVC and VJ height and increase in sprint time after PE are supported by previous investigations [9,14,29]. In our study, one of the indices of muscle performance (KROM) did not change. In contrast to our finding which indicates no statistically significant changes in KROM, Chatzinikolaou *et al.* [16] reported KROM decline for 48 h after PE. However, in accordance with this finding, Tofas *et al.* [14] did not find any significant changes in the KROM following PE. Since the muscle soreness (DOMS) and edema increase after PE [16] and this condition led to a decrease KROM, this limited DOMS (between 1 and 4, Figure 1B) response coincides with the lack of changes in KROM. However, the possible explanation for these discrepancies may be attributed to different modes of KROM assessment.

Strength performance loss after EIMD is one of the common indicators of muscle trauma [7,8,19]. In this study, we measured isometric strength and found incremental decreases in MIVC for the FG and SG. In the previous studies by Tofas *et al.* [14] and Chatzinikolaou *et al.* [16], the authors reported no significant changes in the isometric and isokinetic torque at knee extensors after the PE protocol. The variation in results between these studies could be different modes and knee angles in leg strength assessment. It has been suggested that following PE, voluntary activation might be impaired as a result of increased muscle pain and tenderness, leading to a reduction in neural drive to the muscle [32]. Hence, it is possible that a reduced voluntary effort was manifested as a reduction in maximal strength. In addition, several researchers reported the loss of concentric, isometric and eccentric strength after EIMD [33,34], which would undoubtedly exacerbate the stretch-shortening capabilities of the muscle and result in an increased contact time. The observations in this further confirm that EIMD not only impairs maximal strength but also impairs VJ height.

With regard to VJ height following the PE protocol, it is notable that the AG and SG returned to baseline level at 72 h post-exercise, whereas VJ height remained significantly lower at 72 h for the FG. A number of studies have observed that EIMD causes prolonged reductions in jump performance [6,10,12,16]. The decline in VJ performance is attributed to the inflammatory response to muscle damage [16]. This suggests that damaged muscle has a reduced tolerance to impact forces during movements involving the stretch-shortening cycle, resulting in decreases in VJ height. Impaired muscle glycogen resynthesis following EIMD is also well documented [35]. It is therefore possible that a reduced glycogen content in the knee extensors may have contributed to the decrease in muscle function and power output observed by the decrease in VJ performance. Another possible explanation for the decrement in VJ height after muscle-damaging exercise could be muscle soreness [33], muscular pain experienced as a result of the EIMD-reduced participant’s tolerance to exercise, protecting the muscle against further injury with a reduced neural drive to the muscle [36,37,38]. The disruption of normal neural function following muscle damage has been associated with the subsequent inhibition of muscle function and it has previously been considered that perceptions of soreness may serve as a protective mechanism following damaging exercise to prevent full muscle activation from exacerbating damage and this consequently leads to VJ height decrement [19,37,38].

Studies have also shown a reduced excitation-contraction (E-C) coupling efficiency due to a reduction in calcium release per action potential, which has been associated with a decrease in maximal force production for several days following PE [35,38]. Therefore, a reduction in the force-generating capability due to E-C coupling failure would reduce the ability of the muscle to produce power. It is plausible that repeated stretching of the quadriceps during plyometric jumping may have led to preferential disruption of type II muscle fibers, as a result of early fatigue and temporary increases in muscle stiffness caused within these fibers by the eccentric component of the plyometric exercise [39].

Sprint times in the SG and FG significantly increased at 24 h and recovered to baseline completely at 72 h after PE, whereas no significant change was observed for the AG. These findings are consistent with previous studies [11,40] which reported that linear sprint running time over 10 m was significantly reduced by ~3% following 100 VJs on a rigid surface. In contrast, Semark and coworkers [41] reported that completing 70 drop jumps did not significantly impair 10-m sprint running performance, although evidence of greater muscle damage is presented in the present study. There was no significant change in 10-m sprint time for the AG. It appears that plyometric exercises in an aquatic setting do not seem to impair sprint performance, which may be attributed to the lower-impact forces experienced within the buoyant environment of the aquatic condition.

Overall, the results of this study indicated that PE in water provided a low-impact medium that produced less stress in the lower limbs and the buoyancy properties of the water reduced weight-bearing stress on the limbs and consequently reduced the muscle soreness and damage in addition to lesser decrements in performance capacity [2,4]. Furthermore, the high-shock absorptive qualities of sand could decrease the impact forces experienced during PE, potentially to reduce muscle damage and soreness, in addition to lesser decreases in maximal-intensity exercise performance tests [21,22,42,43]. Therefore, aquatic and sand environments might be considered as viable options for recovery and rehabilitation for the plyometric training sessions.

In contrast to the positive effects of sand and aquatic conditions in reducing muscle damage and soreness following PE, it appears that training on these surfaces has negative effects on SSC [44]. On the other hand, the friction and instability of sand may induce negative effects on SSC, decreases in myotatic reflex, degradation of elastic energy potentiation and an increase in the amortization phase, resulting performance decrements [21,44]. Similarly, water resistance plays an important role in decreasing the properties of SSC by increasing the time period between eccentric-to-concentric muscle action, deficits in SSC capability, and loss of elastic energy resulting in decreases in performance [20]. However, future studies should investigate the safety of prolonged plyometric training on sand and in water, because joint injury and overuse may occur after longitudinal plyometric training. Although the magnitude of muscle damage is less for PE performed on sand and in water than for that on wood surfaces, it does not necessarily mean that sand and aquatic surfaces are preferable for training. Although the preliminary results of the present study suggest that plyometric training on sand and in water may be potentially useful, the biomechanical characteristics of jumping may increase the risk of overuse injuries to the low back. Also, consideration should be given to how the difference in surface affects the training effect. The specific type of exercise being performed should be considered when choosing a training surface, remembering that plyometric drills may require a firm surface.

## 8. Perceived Exertion

The RPE increased progressively from set 1 to set 10 for all treatment groups, These findings are in line with Brown and coworkers [28], who examined the effects of eight sets of 10 repetitions of depth jumps from an 80-cm box on RPE and reported progressive increases in RPE during PE from ~11 to ~17 for college-aged men. In the present study, it is interesting to note that there was no statistical difference between groups in the rate of increase in the RPE, which indicates that the degree of exertion and fatigue experienced within the three conditions was similar.

The current study compared aquatic, sand and firm (wood) surfaces for the development of muscle damage and soreness following EIMD (10 × 10 VJs). Changes in the indirect markers of muscle damage (*i.e.*, CK activity, VJ, sprint, KROM and strength) were generally smaller following the PE in the aquatic setting and sand when compared with that performed on the firm surface. High-impact forces in the order of three to four times the body weight can occur during PE on a rigid surface. These high, transient forces impact the musculoskeletal system and can induce acute muscle soreness, muscle damage and even musculoskeletal injuries. Landing on a compliant sand surface will increase the extent of shock absorbance, and consequently decrease the stress to soft tissues and bones of the lower limbs during PE, compared to the same exercise on rigid surface. An aquatic setting can provide a safer environment in which impact is minimized, but the sense of effort experienced is similar to that experienced doing plyometric activity on dry land. The limitations of this study include the low number of subjects, which prevents generalization of the results. Additionally, the results of the current investigation are based on a non-sporting population, and further research is needed to determine if similar effects are obtained in the sporting population. The results of this investigation therefore suggest that PE performed in an aquatic setting and on a sand surface is less stressful to the muscle-tendon complex than when compared to a firm surface, and this may have implications for rehabilitation and in the prevention of musculoskeletal injury. Thus, it can be recommend that coaches and strength and conditioning professionals use safe surfaces for plyometric exercises and training to decrease symptoms of muscle damage; however, they must remember that plyometric drills may require a firm surface. Moreover, there is no financial support for this project and we did not measure some of the blood indicators of muscle damage, biopsy and MRI, which can be a subject for further research.

## Figures and Tables

**Table 1:** Mean ± SD for rating of perceived exertion after each set of VJ exercise.

Group	Sets									
	1	2	3	4 *	5 ^†^	6	7	8	9 ^‡^	10 ^‡^
AG	8.25 ± 2.3	9 ± 2.3	9.1 ± 2.1	9.7 ± 2	10.8 ± 2.2	10.6 ± 2	11.2 ± 2.1	11.8 ± 1.8	12.7 ± 2.1	13.5 ± 2.1
SG	7.5 ± 0.9	8.5 ± 0.7	9.3 ± 1.1	10.3 ± 1.9	10.8 ± 2.1	11.7 ± 2.6	12.3 ± 2.6	12.8 ± 2.9	13.3 ± 2	14.1 ± 2
FG	7.1 ± 0.8	7 ± 1.4	8.62 ± 1.8	8.8 ± 2.1	9.1 ± 2.4	9.8 ± 2.8	10.5 ± 2.8	11 ± 3	11.2 ± 3.4	12 ± 3.8

AG: Aquatic group; SG: Sand group; FG: Firm group. *: Significant difference compared to set 1 (*p* ≤ 0.05); ^†^: Significant difference compared to set 2 (*p* ≤ 0.05); ^‡^: Significant difference compared to set 7 (*p* ≤ 0.05).

## References

[1] Potach D., Chu D.A., Bachle T., Erale R. (2000). Plyometric training. Essential of Strength and Conditioning.

[2] Arazi H., Asadi A. (2011). The effect of aquatic and land plyometric training on strength, sprint, and balance in young basketball players. J. Hum. Sport Exerc..

[3] Asadi A., Arazi H. (2012). Effects of high-intensity plyometric training on dynamic balance, agility, vertical jump and sprint performance in young male basketball players. J. Sport Health Res..

[4] Robinson L.E., Décor S.T., Merrick M.A., Buckworth J. (2004). The effects of land *vs.* aquatic plyometrics on power, torque, velocity, and muscle soreness in women. J. Strength Cond. Res..

[5] Asadi A., Saez de Villarreal E., Arazi H. (2015). The effects of plyometric type neuromuscular training on postural control performance of male team basketball players. J. Strength Cond. Res..

[6] Turner A.M., Owings M., Schwane J.A. (2003). Improvement in running economy after 6 weeks of plyometric training. J. Strength Cond. Res..

[7] Byrne C., Twist C., Eston R.G. (2004). Neuromuscular function after exercise-induced muscle damage: Theoretical and applied implications. Sports Med..

[8] Eston R.G., Byrne C., Twist C. (2003). Muscle function after exercise-induced muscle damage: Considerations for athletic performance in children and adults. J. Exerc. Sci. Fit..

[9] Jakeman J.R., Byrne C., Eston R.G. (2010). Lower limb compression garment improves recovery from exercise-induced muscle damage in young, active females. Eur. J. Appl. Physiol..

[10] Marginson V., Rowland A.V., Gleeson N.P., Eston R.G. (2005). Comparison of the symptoms of exercise-induced muscle damage after an initial and repeated bout of plyometric exercise in men and boys. J. Appl. Physiol..

[11] Twist C., Eston R.G. (2005). The effects of exercise-induced muscle damage on maximal intensity intermittent exercise performance. Eur. J. Appl. Physiol..

[12] Twist C., Eston R.G. (2007). The effect of muscle-damaging exercise on maximal intensity cycling and drop jump performance. J. Exerc. Sci. Fit.

[13] Twist C., Eston R.G. (2009). The effect of exercise-induced muscle damage on perceived exertion and cycling endurance performance. Eur. J. Appl. Physiol..

[14] Tofas T., Jumurtas A.Z., Fatouros I., Nikolaidis M.G., Koutedakis Y., Sinouris E.A., Papageorgakopoulou N., Theochathios D.A. (2008). Plyometric exercise increases serum indices of muscle damage and collagen breakdown. J. Strength Cond. Res..

[15] Byrne C., Eston R.G. (2002). The effects of exercise-induced muscle damage on isometric and dynamic knee extensor strength and vertical jump performance. J. Sports Sci..

[16] Chatzinikolaou A., Fatouros I.G., Gourgoulis V., Avloniti A., Jamurtas A.Z., Nikolaidis M.G., Douroudos I., Michailidis Y., Beneka A., Malliou P. (2010). Time course of changes in performance and inflammatory responses after acute plyometric exercise. J. Strength Cond. Res..

[17] Chen T.C., Chen H.-L., Wu C.-J., Lin M.-R., Chen C.-H., Wang L.-L., Wang S.-Y., Tu J.-H. (2007). Changes in running economy following a repeated bout of downhill running. J. Exerc. Sci. Fit..

[18] Eston R.G., Peters D. (1999). Effects of cold water immersion on the symptoms of exercise-induced muscle damage. J. Sports Sci..

[19] Miyama M., Nosaka K. (2004). Influence of surface on muscle damage and soreness induced by consecutive drop jumps. J. Strength Cond. Res..

[20] Martel G.F., Harmer M.L., Logan J.M., Parker C.B. (2005). Aquatic plyometric training increases vertical jump in female volleyball players. Med. Sci. Sports Exerc..

[21] Barrett R.S., Neal R.J., Roberts L.J. (1997). The dynamic loading response of surfaces encountered in beach running. J. Sci. Med. Sport.

[22] Impellizzeri F.M., Rampinini E., Castagna C., Martino F., Fiorini S., Wisloff U. (2008). Effect of plyometric training on sand *versus* grass on muscle soreness and jumping and sprinting ability in soccer players. Br. J. Sports Med..

[23] Harriss D.J., Atkinson G. (2011). Ethical standards in sport and exercise science research. Int. J. Sports Med..

[24] Faul F., Erdfelder E., Lang A.G., Buchner A. (2007). G*Power 3, a flexible statistical power analysis program for the social, behavioural and biomedical sciences. Behav. Res. Methods.

[25] Clarkson H.M. (2005). Joint Motion and Function Assessment.

[26] Byrne C., Eston R.G., Edwards R.H.T. (2003). Characteristics of isometric and dynamic strength loss following eccentric exercise-induced muscle damage. Scan. J. Med. Sci. Sports.

[27] Borg G. (1998). Borg’s Perceived Exertion and Pain Scales.

[28] Brown A.G., Ray M.W., Abbey B.M., Shaw B.S., Shaw I. (2010). Oxygen consumption, heart rate, and blood lactate responses to an acute bout of plyometric depth jumps in college-aged men and women. J. Strength Cond. Res..

[29] Davies R.C., Eston R.G., Poole D.C., Rowlands A.V., Dimenna F., Wilkerson D.P., Jones A.M. (2008). The effect of eccentric exercise induced muscle damage on the dynamics of muscle oxygenation and pulmonary oxygen uptake. J. Appl. Physiol..

[30] Macaluso F., Isaacs A.W., Myburgh K.H. (2012). Preferntial type II muscle fiber damage from plyometric exercise. J. Athl. Train..

[31] Macaluso F., Isaacs A.W., di Felice V., Myburgh K.H. (2014). Acute change of titin at mid-sarcomer remains despite 8 wk of plyometric training. J. Appl. Physiol..

[32] Byrne C., Eston R.G. (2002). Maximal-intensity isometric and dynamic exercise performance after eccentric muscle actions. J. Sports Sci..

[33] Hortobágyi T., Houmard J., Fraser D., Dudek R., Lambert J., Tracy J. (1998). Normal forces and myofibrillar disruption after repeated eccentric exercise. J. Appl. Physiol..

[34] Costill D.L., Pascoe D.D., Fink W.J. (1990). Impaired muscle glycogen synthesis after eccentric exercise. J. Appl. Physiol..

[35] Ingalls C.P., Warren G.L., Williams J.H., Ward C.W., Armstrong R.B. (1999). E-C coupling failure in mouse EDL muscle after *in vivo* eccentric contractions. J. Appl. Physiol..

[36] Avela J., Kyrolainen H., Komi P.V., Rama D. (1999). Reduced reflex sensivity persists several days after long-lasting stretch-shortening cycle exercise. J. Appl. Physiol..

[37] Komi P.V. (2000). Stretch-shortening cycle: A powerful model to study normal and fatigued muscle. J. Biomech..

[38] Warren G.L., Lowe D.A., Hayes D.A., Farmer M.A., Armstrong R.B. (1993). Excitation failure in eccentric contraction-induced injury of mouse soleus muscle. J. Appl. Physiol..

[39] Brockett C.L., Morgan D.L., Gregory J.E., Proske U. (2002). Damage in different types of motor units following repeated active lengthening of the medial gastrocnemius muscle of the cat. J. Appl. Physiol..

[40] Highton J.M., Twist C., Eston R.G. (2009). The effects of exercise-induced muscle damage on agility and sprint running performance. J. Exerc. Sci. Fit..

[41] Semark A., Noakes T.D., St Clair Gibson A., Lambert M.I. (1999). The effect of a prophylactic dose of flurbiprofen on muscle soreness and sprinting performance in trained subjects. J. Sports Sci..

[42] Binnie M.J., Dawson B., Pinnington H., Landers G., Peeling P. (2014). Sand training: A review of current research and practical applications. J. Sports Sci..

[43] Binnie M.J., Dawson B., Arnot M.A., Pinnington H., Landers G., Peeling P. (2014). Effect of sand *versus* grass training syrfaces during an 8-week pre-season conditioning programme in team sport athletes. J. Sports Sci..

[44] Mirzaei B., Norasteh A., Saez de Villarreal E., Asadi A. (2014). Effects of 6 weeks of depth jump *vs.* countermovement jump training on sand on muscle soreness and performance. Kinesiology.

